# Evidence of re-osseointegration after electrolytic cleaning and regenerative therapy of peri-implantitis in humans: a case report with four implants

**DOI:** 10.1007/s00784-021-04345-1

**Published:** 2022-03-04

**Authors:** Dieter D. Bosshardt, Urs R. Brodbeck, Florian Rathe, Thomas Stumpf, Jean-Claude Imber, Paul Weigl, Markus Schlee

**Affiliations:** 1 Department of Periodontology, School of Dental Medicine, University of Bern, Bern, Switzerland; 2 Robert K. Schenk Laboratory of Oral Histology, School of Dental Medicine, University of Bern, Bern, Switzerland; 3 Private Practice, Zahnmedizin Zurich Nord, Herzogenmühlestrasse 14, CH-8051 Zürich, Switzerland; 4 Private Practice, Forchheim, Germany; 5 Department of Prosthodontics, Danube University, Krems, Austria; 6 Department of Medical Technology Research, Carolinum University Dental Institute, Faculty of Medicine at J.W. Goethe-University, Frankfurt am Main, Germany; 7 Department of Maxillofacial Surgery, Goethe University, Frankfurt am Main, Germany

**Keywords:** Dental implants, Peri-implantitis, Therapeutics, Re-osseointegration, Electrolytic cleaning

## Abstract

**Objective:**

To evaluate re-osseointegration after electrolytic cleaning and regenerative therapy of dental implants with peri-implantitis in humans.

**Material and methods:**

Four dental implants that developed peri-implantitis underwent electrolytic cleaning followed by regenerative therapy with guided bone regeneration. All four implants developed recurrent peri-implantitis and were therefore explanted 6 to 13 months later. Radiographic bone level, probing depth, and bleeding on probing were determined at the time of surgery, 6 months later, and before implant retrieval. The peri-implant tissues were histologically and histomorphometrically analyzed.

**Results:**

All four implants demonstrated radiographic and histological bone gain, reduced probing depth, and bleeding on probing. Radiographic bone gain was 5.8 mm mesially and 4.8 mm distally for implant #1, 3.3 mm and 2.3 mm for implant #2, 3.1 mm and 0.5 mm for implant #3, and 3.5 mm and 2.8 mm for implant #4. The histometric mean and maximum vertical bone gain for implant #1 to #4 was 1.65 mm and 2.54 mm, 3.04 mm and 3.47 mm, 0.43 mm and 1.27 mm, and 4.16 mm and 5.22 mm, respectively. The percentage of re-osseointegration for implant #1 to #4 was 21.0%, 36.9%, 5.7%, and 39.0%, respectively. In one implant, the newly formed bone was deposited directly onto calculus on the implant surface.

**Conclusions:**

We found that (1) re-osseointegration is possible on a formerly contaminated implant surface and (2) the electrolytic cleaning process seems to be effective enough at sites with calculus residues.

**Clinical relevance:**

Since re-osseointegration can be achieved by electrolytic cleaning, this decontamination technique may be considered as a future treatment concept.

## Introduction

Dental implants have become a predictable and effective treatment option for the replacement of missing teeth in completely and partially edentulous patients. Excellent clinical long-term data exist, with survival rates even exceeding 95% over 10 years [1]. Nevertheless, complications do occur and can compromise implant longevity [2]. Owing to the ever-increasing number of implants placed in the oral cavity, it can be anticipated that the number of complications will increase over time as well.

A frequent biological complication is the development of peri-implantitis, a pathological condition occurring in tissues around dental implants. Peri-implantitis is a biofilm-associated pathological condition, characterized by inflammation in the peri-implant mucosa and subsequent progressive loss of supporting bone [3]. The inflammatory process is caused by bacteria that form a biofilm on the implant surface, resulting in soft tissue degradation, peri-implant pocket formation, and bone resorption. The loss of bone exposes the implant surface and leads to esthetic problems and compromises osseointegration. The prevalence rates of peri-implantitis reported in studies vary greatly due to heterogeneous disease definitions and differences applied for case definitions [2]. A systematic review with meta-analysis reported a weighted mean prevalence of 22% for peri-implantitis, with a range of 1 to 45% [4].

Therapy of peri‐implantitis, followed by regular supportive care, results in high patient‐ and implant‐level survival in the medium to long term [5]. Favorable results were reported after treatment, with clinical improvements and stable peri‐implant bone levels in the majority of patients [5].

To eliminate the inflammation, anti-infective therapy must precede any regenerative treatment. Various modalities to treat peri-implantitis have been tested alone or in combinations in animals and/or in humans [2]. While non-surgical procedures are not very effective, a wide range of surgical protocols are currently applied. The first step of the surgical procedure is flap elevation, followed by soft tissue degranulation. Numerous methods of implant surface decontamination have been tested and applied, including mechanical, sonic, and ultrasonic scalers, lasers, air-powder abrasion, and various chemical solutions such as chlorhexidine digluconate, citric acid, hydrogen peroxide, and saline [2]. Currently, none of the surface decontamination methods has proven to be superior.

Animal studies have shown that re-osseointegration following peri-implantitis treatment is possible with various methods, although to a greatly varying degree [6–10]. Very recently, re-osseointegration of contaminated implant surfaces following electrolytic cleaning, a new decontamination technique, was demonstrated in a preclinical study [11]. The aim of the present study was to evaluate whether the electrolytic cleaning technique can achieve re-osseointegration of dental implants that developed peri-implantitis following placement in humans.

## Materials and methods

This study was registered (BfArM DA/CA99, DIMDI 00,010,977) and approved by the “Ethik-Kommission der Bayerischen Landesärztekammer” (BASEC_No. DE/EKBY10) with the registration code 17,075. Oral and written description of the study, including the risks, benefits, and alternative therapies, were provided to all patients. All patients signed a written informed consent form based on the Helsinki Declaration of 1997, which was revised in 2000, before they were implemented in the study. Four implants from three patients were included in this case series (Table 1). Two patients with three implants were originally participating in the registered study. The third patient was treated in the dental practice of the authors in Forchheim, Germany, and was not included in the registered study, but signed the written informed consent.

**Table 1:** Specimen overview

Implant Number	Patient Gender Birth Date	Implant Type	Implant Position	Implantation Date	Electrolytic Therapy Date	Regenerative Therapy	Explantation Date	Period between Electrolytic Therapy and Explantation
#1	Female September 25, 1963	Steri Oss HA-coated 3,8/12mm	37	January 23, 1995	October 16, 2018	Bio-Oss® + autogenous bone (50:50) + Bio-Gide®	April 9, 2019	6 months
#2	Female February 3, 1956	Straumann BL, RC, SLActive 4,1/12mm	15	September 22, 2011	October 18, 2018	Bio-Oss® + autogenous bone (50:50) + Bio-Gide®	November 25, 2019	13 months
#3	Female February 3, 1956	Straumann BL, RC, SLActive, Ti 4,8/12mm	16	September 22, 2011	October 18, 2018	Bio-Oss® + autogenous bone (50:50) + Bio-Gide®	November 25, 2019	13 months
#4	Female April 7, 1961	Straumann BL, RC, SLActive, Ti 4,1/14 mm	11	December 12, 2017	April 8, 2019	maxgraft® + autogenous bone + A-PRF + Jason® membrane	January 27, 2020	9 ½ months

The four implants developed peri-implantitis and were subsequently subjected to suprastructure removal and electrolytic cleaning. The electrolytic device and its mode of action are described elsewhere [12]. Briefly, an electrical current is applied to an implant sprayed with a sodium formiate solution. The current dissociates the water into hydrogen anions (OH^−^) and cations (H^+^). Hydrogen ions interact with the captured electrons to form hydrogen bubbles that lift the biofilm off the implant surface.

In addition to the electrolytical decontamination process, regenerative therapy was performed. This consisted of guided bone regeneration (GBR) using a mixture of a bone substitute and autogenous bone particles covered with a barrier membrane (Table 1). For three implants, a xenogeneic bone substitute (Bio-Oss®, Geistlich Pharma AG, Wolhusen, Switzerland) and a resorbable membrane (Bio-Gide®, Geistlich Pharma AG, Wolhusen, Switzerland) were used. In one implant, an allogeneic bone substitute (maxgraft®, Botiss Biomaterials GmbH, Zossen, Germany) and a resorbable membrane (Jason® membrane, Botiss Biomaterials GmbH, Zossen, Germany) were used. If the bone defect was not contained, umbrella screws (Ustomed, Tuttlingen, Germany) were used to tent up the space and to immobilize the bone fillers (Implant #1 received two umbrella screws, implant #2 three umbrella screws, implant #3 three umbrella screws, and implant #4 no umbrella screws).

**Fig. 1:**
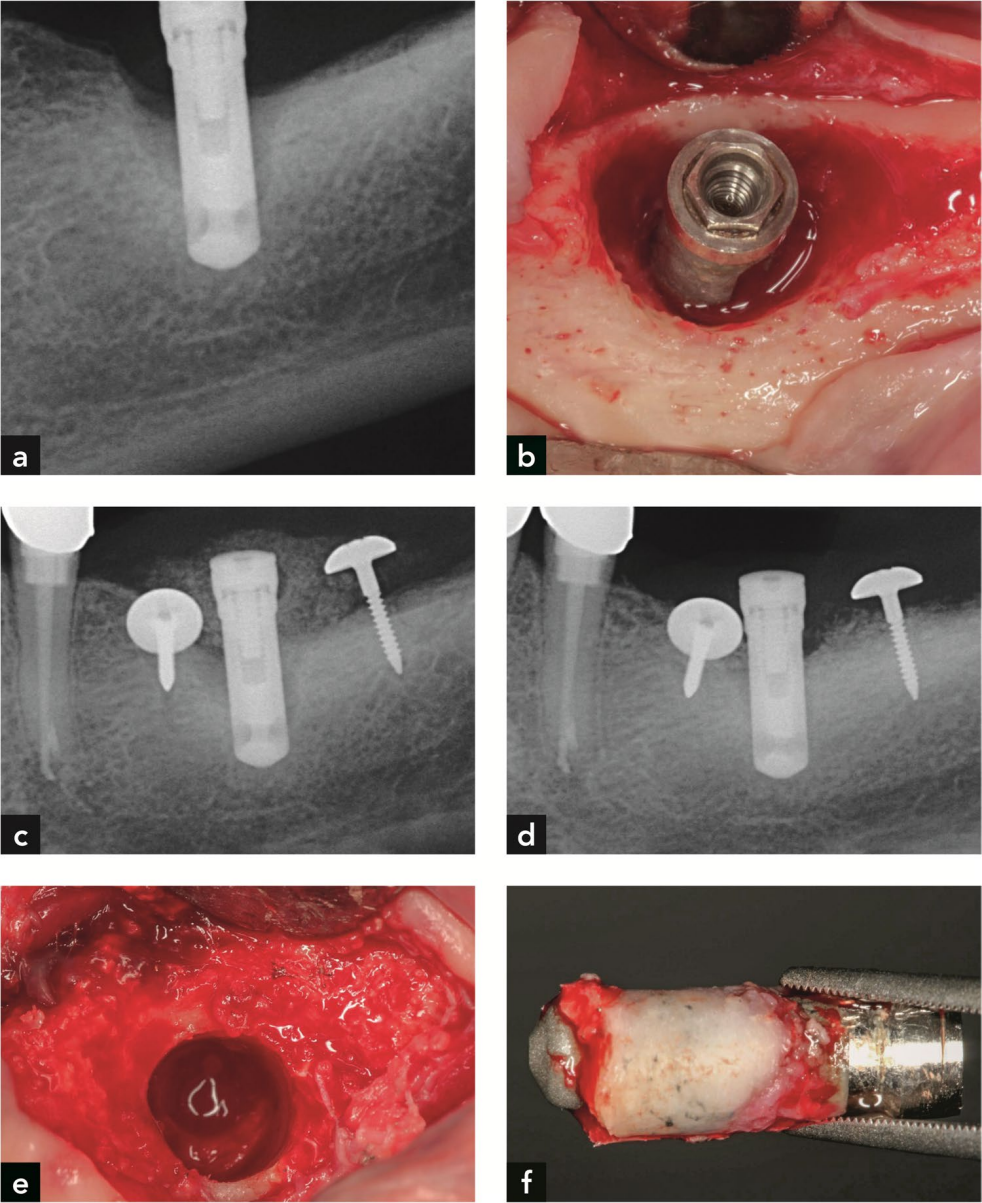
Implant #1. (**a**) Radiographs demonstrating peri-implant bone loss on the day of electrolytic treatment. (**b**) Implant and bone after debridement and electrolytic cleaning. (**c**) Radiograph after GBR with the use of umbrella screws to tent up the non-contained bone defect. (**d**) Radiograph on the day of explantation. (**e**) Resident bone after implant retrieval with a trephine bur, and (**f**) Retrieved implant with surrounding bone

 Submerged healing and reinstallation of suprastructures 6 months after surgery were planned for all four implants. A dehiscence developed around all of them within the first three weeks after surgery, leading to implant exposure. During the follow-ups, recurrent peri-implantitis was diagnosed in all four cases. Explantation was carried out with a trephine bur with a diameter slightly larger than the implant diameters (Fig. 1f). Thereafter, histological and histomorphometric evaluation was performed. The periods between electrolytic therapy and explantation varied between 6 and 13 months (Table 1).

Peri-implantitis was diagnosed according to a definition described in a Consensus Report of a working group at the 2017 World Workshop on the Classification of Periodontal and Peri-Implant Diseases and Conditions [3]. Since all cases were referred patients, no clinical and radiographic baseline data were available. Consequently, the definition of peri-implantitis in the absence of baseline data was applied (i.e. radiographic evidence of bone level ≥3 mm and/or probing depths ≥6 mm in conjunction with profuse bleeding represents peri-implantitis [13]). Radiographs, probing depth (PD), and bleeding on probing (BoP) were assessed before surgery (T0), after 6 months (T1) and before explantation (T2) at six points (m, mb, b, db, d, dl) using a periodontal probe with a 1-mm scale (PCPUNC 15, HuFridy, Chicago, IL, USA). The distance from the implant platform to the most apical position of bone (P-B) was assessed as described previously at the same six points, as PD and BoP were measured using the described periodontal probe [12]. The peri-implant bone defect anatomy during surgeries were classified with the use of the RP (regenerative potential) classification according to Schlee and coworkers [12]. All the clinical assessments were performed by one examiner (MS). Radiographs were taken in a rectangular manner using a Rinn Holder and assessed by the author TS using software (DBS Win, Dürr, Bietigheim-Bissingen, Germany). Potential failures in vertical dimension were corrected by calibrating the software with the known length of the implant.

### Histological processing

The implants and their surrounding tissues were fixed in 10% buffered formalin, dehydrated in an ethanol series, and embedded in methylmethacrylate (MMA). Undecalcified ground sections of 600 µm thickness were cut parallel to the long axis of the implants using a low-speed diamond saw with coolant (Varicut® VC-50, Leco, Munich, Germany). The first section of each implant was produced in the center axis of the implant. All following sections were produced at a right angle to the central section. This way, a larger number of sections, covering a larger implant surface area, were produced. After mounting onto acrylic glass slabs, the sections were ground and polished to a final thickness of about 150 µm (Knuth-Rotor-3, Struers, Rodovre/Copenhagen, Denmark). These ground sections were stained with toluidine blue and basic fuchsin. High-resolution digital photography was performed using a digital camera (AxioCam MRc; Carl Zeiss, Oberkochen, Germany) connected to a microscope (Axio Imager M2; Carl Zeiss).

### Histomorphometry

The base of the former bone defect was determined by the demarcation line interfacing with old bone in the apical part of the implants and newer bone in the implant’s more coronal part. Compared to new bone, old bone is characterized by the presence of secondary osteons, a higher number of cement lines, less residual woven bone, fewer osteocytes, and lighter staining. The vertical distance between this demarcation line and the most coronal bone on the implant surface (fBIC) was measured. In addition, the vertical distance between the most apical bone substitute particle embedded in bone was measured, since this site was an irrefutable sign of new bone development. Furthermore, the vertical distance from the implant shoulder to the fBIC was measured as well. The percentage of newly formed bone, respectively re-osseointegration, was calculated from the base of the former bone defect to the fBIC [14]. 

## Results

### Clinical documentation 

As an example, the clinical documentation of implant #1 is shown in Fig. 1. After detection of peri-implantitis (Fig. 1a), debridement was carried out, followed by electrolytic cleaning (Fig. 1b) and a GBR augmentation procedure with Bio-Oss® and autogenous bone particles (50:50) and a barrier membrane was performed (Fig. 1c). Additionally, two umbrella screws were used to tent up the membrane and immobilize the bone graft. Six months later, recurrent peri-implantitis was diagnosed (Fig. 1d) and the implant was explanted using a trephine bur (Figs. 1e,f).

The changes in radiographic bone height as well as PD values are described in Table 2. An increase in radiographic bone from T0 to T1 was evident for all four implants, whereas vertical bone loss was noticed between T1 and T2. Nevertheless, a radiographic bone gain was measured for all implants over the entire observation period. The mesial and distal bone gain was 5.8 mm and 4.8 mm for implant #1, 3.3 mm and 2.3 mm for implant #2, 3.1 mm and 0.5 mm for implant #3, and 3.5 mm and 2.8 mm for implant #4. A reduction in PD from T0 to T2 was found for all implants. For implant #1, an acute inflammation with BoP and pus formation was found 6 months after surgery. Consequently, recurrent peri-implantitis was diagnosed and no suprastructure was reinstalled. For implants #2 and #3 (from the same patient), incomplete bone fill without BOP and/or pus formation was assessed after a healing period of 6 months and the suprastructures were reinserted. A reassessment of these two implants revealed BoP 9 months after surgery. After 12 months, pus formation and BoP were observed and recurrent peri-implantitis was diagnosed. In the case of implant #4, the suprastructure was reinserted 6 months after surgery despite the fact of incomplete bone fill and BoP. After 9 months, a progredient bone loss was observed in conjunction with BoP and recurrent peri-implantitis was diagnosed. A statistical analysis was not possible due to the small sample size.

**Table 2:** Radiographic and clinical findings

Radiographic findings														
Number of Implant	Location	P-B at T0 in mm		P-B at T1 in mm						P-B at T2 in mm				
		m	d	m			d			m		d		
#1	37	9.8	10.3	4.0			5.5			4.0		5.5		
#2	15	7.6	6.1	4.0			3.6			4.3		3.8		
#3	16	7.1	3.0	3.4			1.1			4.0		2.5		
#4	11	9.4	9.0	5.2			4.8			5.9		6.2		
Clinical findings														
Number of Implant	Location	RP Classification	PD at T0 in mm						PD at T2 in mm					
			db	b	mb	ml	l	dl	dl	b	mb	ml	l	dl
#1	37	RP 2 defect	9	11	9	8	8	7	6	6	6	5	5	6
#2	15	RP 3 defect	7.5	7	7	8	8	7.5	3	3	3	3	3	3
#3	16	RP 3 defect	8	8.5	8	9	9	8	4.5	4	4	3	4	4
#4	11	RP 2 defect	9	9	9	9	8	9	4	4	3	2	3	4

*Note: P-B* distance from the implant platform to the most apical position of bone; *RP* (*regenerative potential*) *classification* bone defect anatomy at T0; *PD* probing depth; *mm* millimeter; *db* disto-buccal; b buccal; *mb* mesio-buccal; *ml* mesio-lingual; *l* lingual; *dl* disto-lingual; *T0* time point before surgical treatment; *T1* 6 months after surgery; *T2* time point before explantation

### Histological and histomorphometric evaluation.

The histomorphometrical data are presented in Table 3.

#### Implant #1

**Fig. 2:**
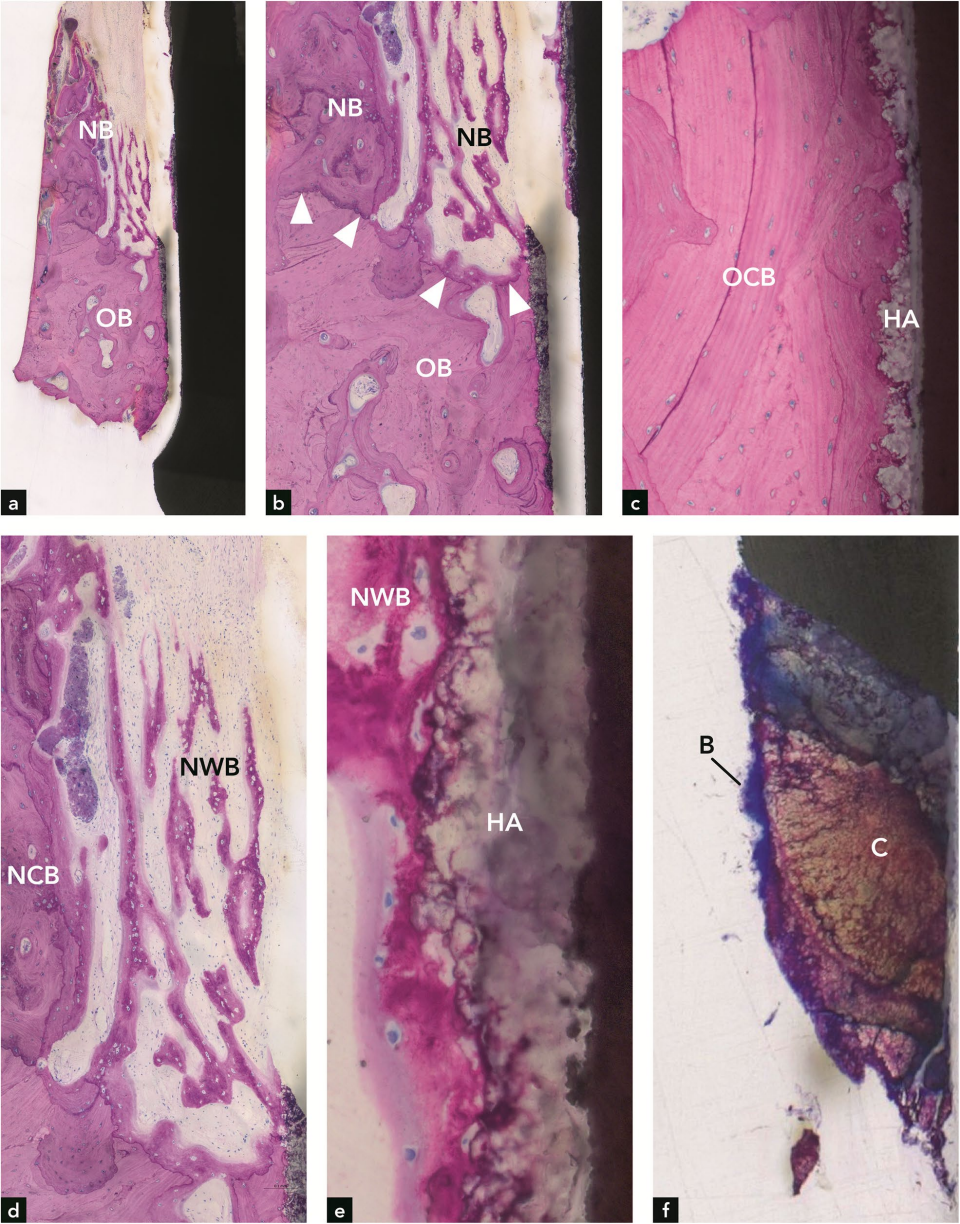
Implant #1. (**a**,**b**) Bone in the apical half of the implant with a demarcation line (arrowheads) between old bone (OB) and new bone (NB). (**c**) Old, compact bone (OB) in contact with the HA coating on the implant. (**d**) Higher magnification of (**a**) illustrating new compact bone (NCB) and new woven bone (NWB) coronal to the defect bottom. (**e**) New woven bone on the HA coating of the implant. (**f**) Calculus (**C**) with biofilm (**B**) on the coronal implant surface

It was possible to distinguish between older and newer bone (Fig. 2a,b). The older bone consisted of lamellar bone that contained secondary osteons, whereas the newer bone was either lamellar with primary osteons or trabecular, consisting of woven bone. In the apical part, there was mature compact (lamellar) bone in direct contact with the HA coating on the implant (Fig. 2c). In other, yet still apical regions, this direct contact was also observed, but the HA coating was detached from the implant surface (Fig. 2b). A demarcation line separated old apical bone from new coronal bone (Fig. 2b). The new bone away from the implant surface was compact and mainly made up of primary osteons, whereas the bone closer to and in contact with the HA-coated implant had a trabecular structure and consisted of woven bone (Fig. 2d,e). In the very coronal exposed part of the implant, calculus (C) deposits with or without a biofilm coating were observed on the implant surface (Fig. 2f). The calculus had an uneven contour and reddish to brownish stain, whereas the biofilm had a dark blue color and a filamentous surface structure. The maximum vertical distance from the interface between old and new bone to the most coronal bone on the implant surface was 2.54 mm (mean = 1.65 mm). The minimum vertical distance from the implant shoulder to the most coronal new bone on the implant surface was 6.4 mm (mean = 6.21 mm). The percentage of re-osseointegration was 21.0%.

**Table 3:** Histomorphometric results

Number of Implant	Number of sections	Implant length (mm) mean, SD	Vertical bone gain (mm) mean, SD	Vertical bone gain (mm) maximum	Re-osseointegration (percentage)	IS-fBIC(mm) mean, SD	IS-fBIC (mm) minimum
1	3	11.46 ± 0.40	1.65 ± 0.80	2.54	21.0	6.21 ± 0.45	5.69
2	4	12.30 ± 0.09	3.04 ± 1.05	3.47	36.9	5.19 ± 0.33	4.60
3	5	12.30 ± 0.16	0.43 ± 0.46	1.27	5.7	7.20 ± 0.46	6.63
4	4	14.31 ± 0.11	4.16 ± 0.95	5.22	39.0	6.50 ± 0.40	6.01

*mm* millimeter; *SD* standard deviation

#### Implant #2

**Fig. 3:**
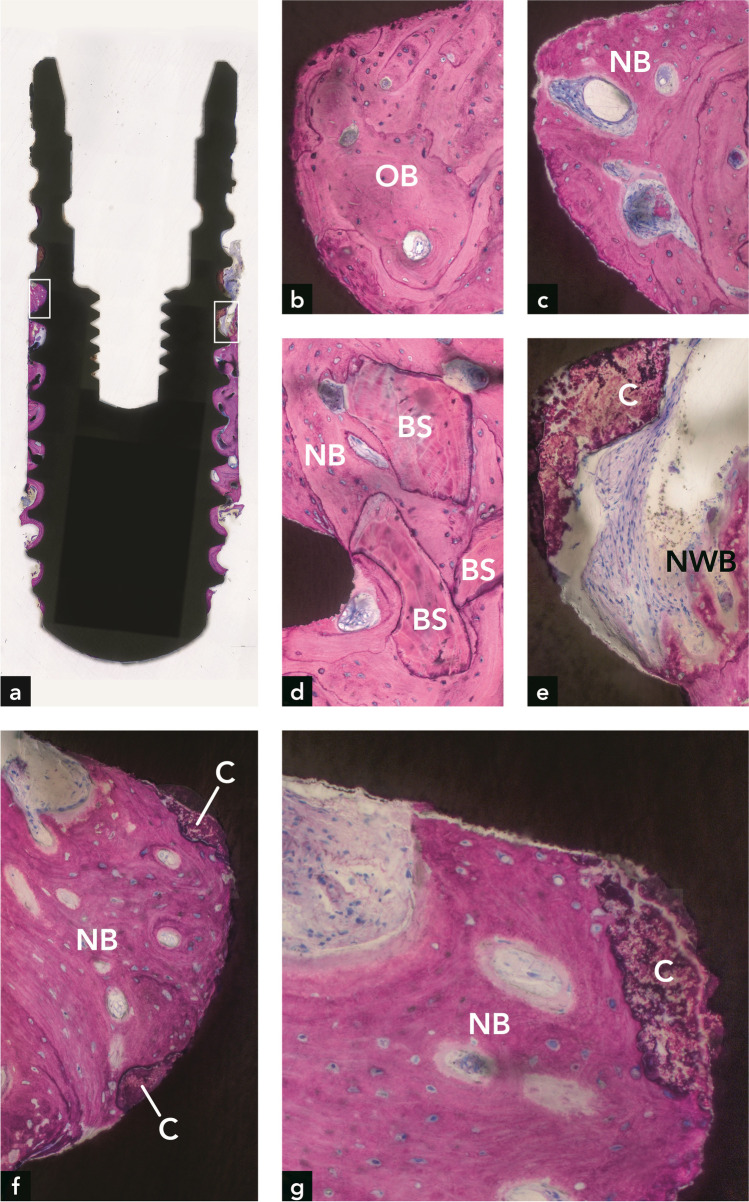
Implant #2. (**a**) Plenty of bone is seen around the implant. (**b**) Old bone (OB) is present in the apical part of the implant. (**c**) New bone (NB) is present in the coronal part of the implant. (**d**) Three bone substitute (BS) particles are embedded in new bone. (**e**) Higher magnification of the right rectangle in (**a**), illustrating new woven bone (NWB) and calculus (**C**) at the coronal termination of bone on the implant surface. (**f**) Higher magnification of the left rectangle in (**a**), illustrating new bone in direct contact with two calculus deposits on the implant surface. (**g**) Higher magnification of direct contact between new bone and calculus in an adjacent section

Bone was observed along a considerable portion of the implant surface (Fig. 3a). In the apical part of the implant, the bone was older and more mature (Fig. 3b) than in the coronal part (Fig. 3c). Although no clear demarcation line between old and new bone was found, the inclusion of Bio-Oss® in the coronal bone indicated that this bone was formed after the electrolytic cleaning (Fig. 3d). Woven bone formation was observed in some locations in the coronal part (Fig. 3e). Calculus was present on the implant surface coronal to the bone on the implant (Fig. 3e). In addition, two calculus deposits were completely covered with newly formed bone (Fig. 3f,g). The new bone at these sites was vital and in direct contact with the calculus. Neither a gap nor an interfacial biofilm layer was present. The absence of inflammation and presence of osteocytes in the bone lacunae indicated bone vitality. The maximum vertical distance between the apical-most Bio-Oss® particle embedded in new bone and the coronal-most bone on the implant surface was 2.6 mm (mean = 1.96 mm). The maximum vertical distance from the interface between old bone and new bone to the coronal-most bone on the implant surface was 3.47 mm (mean = 3.04 mm). The minimum vertical distance from the implant shoulder to the coronal-most point of newly formed bone on the implant surface was 4.6 mm (mean = 5.19 mm). The percentage of re-osseointegration was 36.9%.

#### Implant #3

**Fig. 4:**
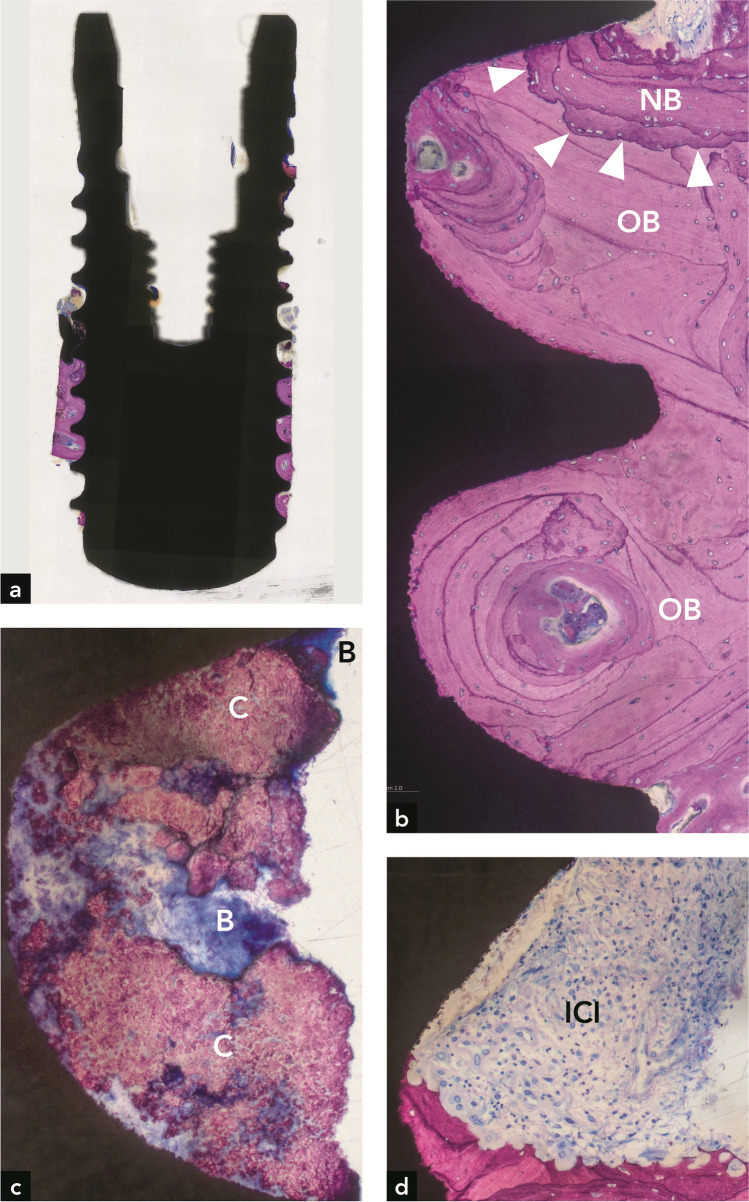
Implant #3. (**a**) Bone is restricted to the apical third of the implant. (**b**) Most peri-implant bone is old bone (OB). Very little new bone (NB) is found at the coronal termination of bone on the implant; (**c**) Calculus (**C**) and biofilm (**B**) are present on the more coronal implant surface; (**d**) In other sections, bone resorption and an inflammatory cell infiltrate (ICI) are present where bone ends on the implant surface

Compared to implant #2, the bone level around implant #3 was significantly more apical (Fig. 4a). Only minute amounts of new bone were observed. Most of the peri-implant bone was old, compact (lamellar) bone, as indicated by the presence of secondary osteons and numerous cement lines (Fig. 4b). The presence of an inflammatory cell infiltrate and resorption cavities (Howship’s lacunae) at the bone crest indicated an active phase of inflammation and bone resorption (Fig. 4d). Calculus and biofilm were observed more coronally on the implant surface (Fig. 4c). Histomorphometry revealed a maximum vertical bone gain of 1.27 mm (mean = 0.43 mm). The minimum vertical distance from the implant shoulder to the coronal-most point of newly formed bone on the implant surface was 6.63 mm (mean = 7.20 mm). The percentage of re-osseointegration was 5.7%.

#### Implant #4

**Fig. 5:**
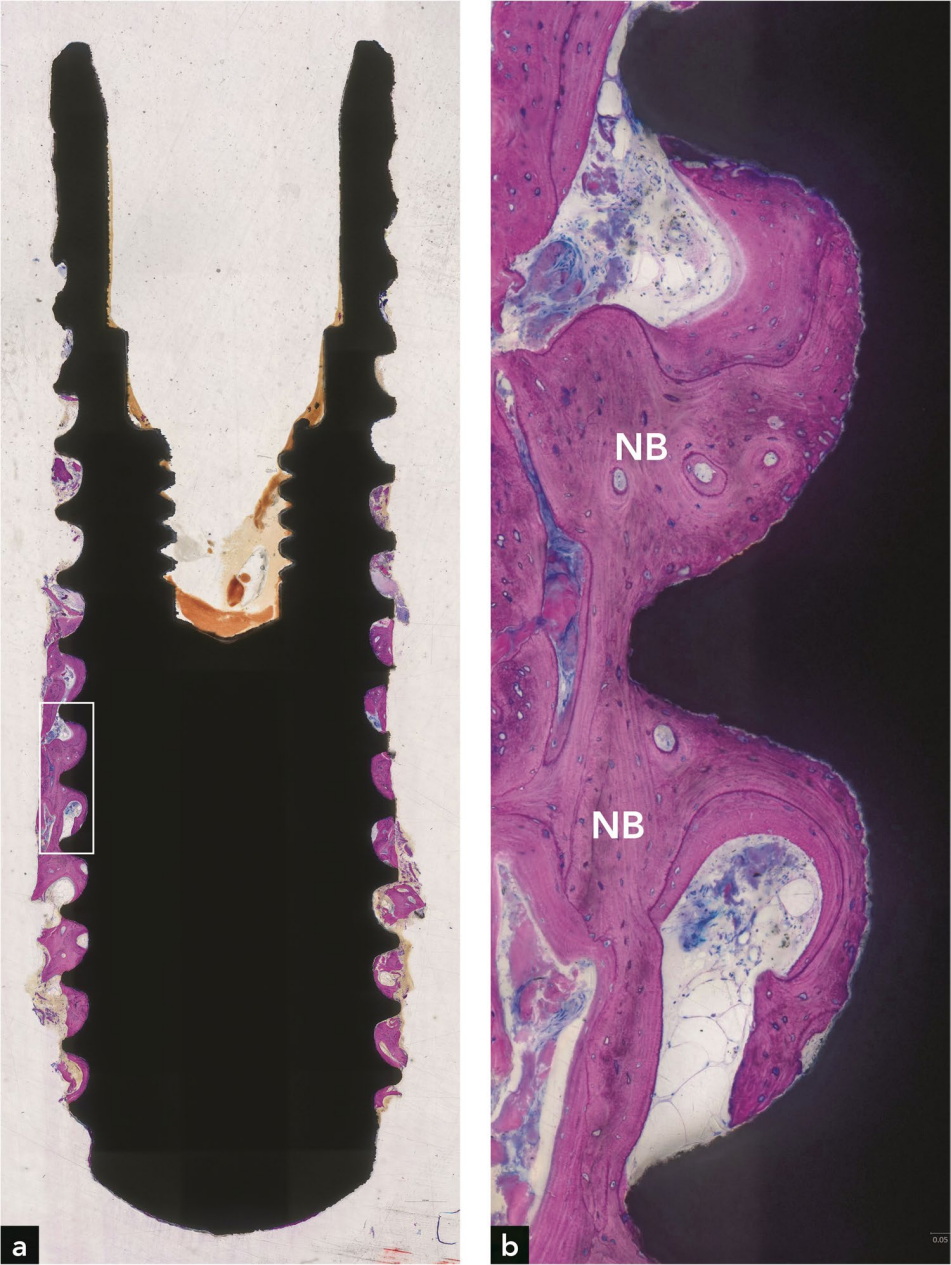
Implant #4. (**a**) Bone covers 50% of the implant length. (**b**) Higher magnification of the rectangle in (**a**), illustrating new bone (NB) stretching along a major portion of the apical half of the implant

More than half of the implant length was covered with bone, and the implant was well osseointegrated (Fig. 5a). The bone consisted mainly of old compact (lamellar) bone, as indicated by the presence of secondary osteons and many cement lines (Fig. 5b). Little woven bone was present in the coronal part of the bone around the implant. Very coronally, far above the bone level, some calculus deposits with or without superficial biofilm were observed. The maximum vertical distance from the interface between old and new bone to the coronal-most bone on the implant surface was 5.21 mm (mean = 4.15 mm). The minimum vertical distance from the implant shoulder to the coronal-most point of newly formed bone on the implant surface was 6.01 mm (mean = 6.58 mm). The percentage of re-osseointegration was 39.0%.

## Discussion

This study describes the clinical, radiographic, histological, and histometric findings of bone around four implants after electrolytic cleaning, which was part of an anti-infective and regenerative peri-implantitis therapy. Originally, removal of these implants was not planned. However, since all four implants became exposed and developed recurrent peri-implantitis, it was indicated to explant them. After explantation, histological and histometric evaluation was performed. Radiographic bone gain and the reduction of pocket depths could be observed for all implants. Moreover, all four implants demonstrated new bone formation, to highly varying degrees. The maximum vertical gain of new bone, as determined histometrically, was 2.54 mm around implant #1, 3.47 mm around implant #2, 1.27 mm around implant #3, and 5.21 mm around implant #4. Taking into account that during the period of recurrent peri-implantitis, any newly formed bone may have become resorbed, more vertical bone gain likely occurred after the electrolytic cleaning and regenerative therapy. In a recent clinical study, significant clinical bone fill, as determined by periodontal probing and standardized radiographs, was observed for all implants [12].

In the present study, the histometric data showing bone gain around implant #1 (maximum of 2.54 m; mean of 1.65 mm) were below those determined by radiography (5.8 mm mesial and 4.8 mm distal). This may have to do with the observed coronal deviation of new bone from the implant surface, which resulted in a more coronal radiographically determined bone height that is unrelated to the bone height directly on the implant surface (Fig. 2a). Regarding the distance from the implant shoulder to the most apical position of the bone, there was a clear inconsistency between radiography and histometry. In three implants, radiography, as opposed to histometry, clearly underestimated this distance. This discrepancy corroborates findings from other studies [15, 16]. Another explanation is that the radiographic bone level varied between distal and mesial sites, and the histological ground sections were not oriented in mesio-distal and oral-vestibular cutting directions. Regarding implant #2, the histometrically determined maximum bone gain of 3.47 mm (mean of 3.04 mm) correlated well with the radiographically determined vertical bone gain of 3.3 mm at the mesial site. Implant #3 had the least histometric bone gain (maximum of 1.27 mm, mean of 0.43 mm). These values correspond well with the low radiographic bone gain of 0.5 mm at the distal site. In implant #4, the histometric bone gain of 5.22 mm (maximum) and 4.16 mm (mean) was greater than the mesial and distal radiographic bone gain of 3.5 mm and 2.8 mm, respectively. In conclusion, in 3 out of 4 implants, the radiographic bone gain exceeded the bone gain determined by histometry.

Regarding the incongruity between radiographic and histometric bone level values, it is vital to note that histological analyses of implants retrieved from humans have their limitations. Compared to animal experiments, where block biopsies display all peri-implant soft and hard tissues in their full dimensions and quality, only a thin bone rim adjacent to the implant can be harvested with the trephine bur in human experiments. In addition, the implant surface and bone can be destroyed if the trephine bur is decentered. Implant #3 (Fig. 4a) and implant #4 (Fig. 5a) clearly show that the trephine bur has touched the implant thread crests. Furthermore, the trephine bur creates shear forces that can lead to artifacts, including separation of bone from the implant surface [17]. Removal of the implant with surrounding tissue in the trephine bur leads to a traumatic separation from the surrounding bone in the apical part, which may result in a complete detachment of bone from the peri-apical implant portion. This can be clearly seen in all four implants (Figs. 2a, 3a, 4a, 5a). In some cases, these artifacts may hinder precise histometric evaluation.

Calculus and biofilm were found on the coronal surface of all four implants, a finding that is not surprising and is consistent with the development of recurrent peri-implantitis. However, a highly unsuspected and novel finding was that on one implant, calculus deposits were found where the implant was enclosed by new bone, which was vital and in direct contact with the calculus. This indicates that the electrolytic cleaning effectively decontaminated the previously contaminated implant surface and allowed bone to be deposited onto the now sterile calculus. In analogy to this novel observation on dental implants, Listgarten and Ellegaard [18] conducted an animal study and found that detoxification of the tooth root surface with 2% chlorhexidine gluconate enabled junctional epithelial cells to attach to calculus/biofilm via a basal lamina and hemidesmosomes. Complete sterilization of biofilm-contaminated implant surfaces by means of electrolytic cleaning has recently been demonstrated in vitro [19]. Although new bone formation on calculus following electrochemical cleaning was observed in the present report, it is important to emphasize that clinicians cannot neglect the decontamination of the implant surface. The regular removal of biofilm and calculus remains an integral part of peri-implantitis therapy.

Re-osseointegration of previously contaminated implant surfaces has been demonstrated in numerous preclinical studies. A large number of decontamination methods were used in these studies, and the amount of newly formed bone varied to a great degree [6–10]. In one preclinical study, re-osseointegration of contaminated implant surfaces was demonstrated following electrolytic cleaning [11], supporting our findings. The pioneering results of the present study demonstrate that re-osseointegration of previously contaminated implant surfaces after electrolytic cleaning is also possible in humans and even in clinical settings, after regenerative therapy for peri-implantitis.

The unfortunate exposure of all four implants was certainly a highly undesirable outcome. It is possible that this event was associated with the vertical augmentation procedure and flap closure. Future applications of this technique need to address this aspect so that proper case selection, including favorable defect anatomy, can be established. Owing to the fact that the regenerative potential for horizontal bone defects is very low as opposed to multi-wall defects [20], the results of the present study are certainly encouraging.

## Conclusions

This histologic case series has proven re-osseointegration of dental implants in humans after electrolytic cleaning and regenerative therapy of peri-implantitis. New bone was even formed on calculus, indicating the electrolytic cleaning process may have an adequate decontaminating effect.
